# Mediterranean Fin Whales (*Balaenoptera physalus*) Threatened by Dolphin MorbilliVirus

**DOI:** 10.3201/eid2202.150882

**Published:** 2016-02

**Authors:** Sandro Mazzariol, Cinzia Centelleghe, Giorgia Beffagna, Michele Povinelli, Giuliana Terracciano, Cristiano Cocumelli, Antonio Pintore, Daniele Denurra, Cristina Casalone, Alessandra Pautasso, Cristina Esmeralda Di Francesco, Giovanni Di Guardo

**Affiliations:** University of Padova, Padua, Italy (S. Mazzariol, C. Centelleghe, G. Beffagna, M. Povinelli);; Istituto Zooprofilattico Sperimentale del Lazio e della Toscana, Rome, Italy (G. Terracciano, C. Cocumelli);; Istituto Zooprofilattico Sperimentale della Sardegna, Sassari, Italy (A. Pintore, D. Denurra);; Istituto Zooprofilattico Sperimentale del Piemonte, Liguria e Valle d’Aosta, Turin, Italy (C. Casalone, A. Pautasso);; University of Teramo Faculty of Veterinary Medicine, Teramo, Italy (C.E. Di Francesco, G. Di Guardo)

**Keywords:** Morbillivirus, dolphin morbillivirus, Balaenoptera physalus, viruses, Mediterranean Sea, fin whales, Mediterranean fin whales, outbreak, morbillivirus infection, nucleoprotein gene, phosphoprotein gene, hemagglutinin gene

## Abstract

During 2011–2013, dolphin morbillivirus was molecularly identified in 4 stranded fin whales from the Mediterranean Sea. Nucleoprotein, phosphoprotein, and hemagglutinin gene sequences of the identified strain were highly homologous with those of a morbillivirus that caused a 2006–2007 epidemic in the Mediterranean. Dolphin morbillivirus represents a serious threat for fin whales.

Fin whales (*Balaenoptera physalus*) living in the Mediterranean Sea belong to a population that is part of the Atlantic stock (*1*). For feeding purposes, these whales tend to concentrate in specific areas, one of which is Pelagos Sanctuary, the widest protected marine area for sea mammals in the Mediterranean Basin between Italy, France, and Monaco. In Pelagos Sanctuary and, in general, in the entire Mediterranean Sea, fin whales are considered vulnerable because of several anthropogenic threats, the most common of which are ship strikes, chemical pollution, and noise (*2*–*4*).

Postmortem investigations on well-preserved whale carcasses are necessary to gain evidence-based insight into the effect these threats have on fin whales; thus, the carcasses of all large cetaceans found stranded along the Italian coastline are systematically examined to determine the cause of death. Because morbillivirus infections have been detected during these postmortem investigations, we conducted a study to determine the effect of this natural threat on the Mediterranean fin whale population.

## The Study

**Figure 1:**
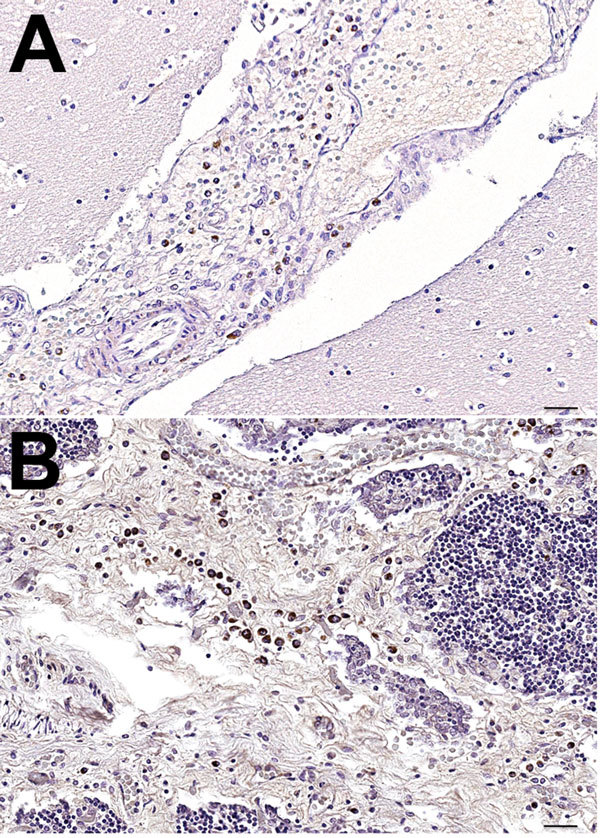
Mayer hematoxylin counterstained tissue samples from a newborn fin whale stranded off the Mediterranean Sea, October 2013. A) Brain tissue showing positive immunostaining for *morbillivirus*. antigen in macrophages in the meningeal space. B) Fin whale thymus showing positive immunostaining for *morbillivirus* antigen in thymocytes and macrophages. For both samples, *morbillivirus* was detected by immunohistochemical analysis, using a rabbit hyperimmune anti–rinderpest virus serum (provided by Pirbright Institute, Pirbright, UK) (*10*). Original magnification ×40. Scale bars indicate 50 μm.

During 2006–2014, a total of 23 fin whales were found stranded along the coast of Italy. We systematically conducted full necropsies and microscopy- and molecular-based analyses on 9 (39%) carcasses that were in good conservation status. Of these 9 fin whales, 2 were juveniles and 2 were calves found during January 2011–February 2013 along the coasts of Tuscany, Sardinia, and Liguria, Italy. These young whales showed pathologic, immunohistochemical (IHC), biomolecular, and/or serologic evidence of dolphin morbillivirus (DMV) infection (Table 1) (*5*–*9*); however, not all of these cases were spatially or temporally related to known fatal DMV-associated outbreaks that occurred during 2006–2014 (Technical Appendix Figure 1). Moreover, in October 2013, a newborn male fin whale was found stranded alive on Elba Island (Tuscany); the whale died after a few hours, and postmortem investigations conducted within 24 hours of death yielded biomolecular and IHC evidence of DMV infection. Viral genome, antigen, or both were found in several tissues, along with a parasitic infection and a generalized lymphocytic depletion. Hyperimmune rabbit anti–rinderpest virus serum (provided by Pirbright Institute, Pirbright, UK) (*10*) was used to detect morbillivirus antigens; only circulating monocytes and tissue macrophages in brain and thymus stained positively (Figure 1).

**Table 1:** Demographic data and examination results for 5 *Dolphin morbillivirus*–positive fin whales stranded off the Mediterranean Sea, 2011–2013*

Whale no., sex/length, m†	Stranding date	Closest DMV outbreak	Dolphin morbillivirus–positive samples, by test		
			RT-PCR‡	IHC analysis‡	VN
1, M/17	2011 Oct 25	Dec 2010–Jun 2011	Liver, spleen, lungs	ND	ND
2, F/10	2011 Nov 3	Dec 2010–Jun 2011	Liver, spleen, lymph nodes, skeletal muscle	Negative	ND
3, M/10	2011 Nov 20	Dec 2010–Jun 2011	Negative	ND	Positive
4, F/15	2013 Mar 19	Jan–Apr 2013	Lymph nodes	ND	Negative
5, F/5	2013 Oct 11	Jan–Apr 2013	Brain, lungs, spleen, thymus	Brain, thymus	Negative

*IHC, immunohistochemical; ND, not done; RT-PCR, reverse transcription PCR; VN, virus neutralization †Whales 1 and 4 were juveniles; whales 2 and 3 were calves. ‡Tissues routinely examined for morbillivirus: lung, liver, spleen, kidney, prescapular and pulmonary lymph nodes, brain, thymus (if available), tonsils, and skeletal muscle.

DMV genome was detected in brain, lung, spleen, and thymus from the newborn whale; viral RNA was extracted from these tissues by using TRIzol reagent (Thermo Fisher Scientific, Waltham, MA, USA). Primer DMV2 (*11*) and the RevertAid First Strand cDNA Synthesis Kit (Thermo Scientific) were used to synthesize cDNA; primer and viral RNA were incubated at 42°C for 60 min followed by 70°C for 5 min. Amplification was performed by using primers DMV-N1 and DMV-P2 (*11*) and Phusion Hot Start II DNA Polymerase (Thermo Scientific) with the following PCR conditions: 30 s at 98°C; 35 cycles of 10 s at 98°C, 30 s at 62°C, 1 min at 72°C; and 10 min at 72°C. Next, the DNA fragments obtained from lung and cerebral cDNA were purified, cloned into the plasmid vector pCR-Blunt II-TOPO (Invitrogen, Life Technologies, Carlsbad, CA, USA) according to the manufacturer’s instructions, and then sequenced.

**Figure 2:**
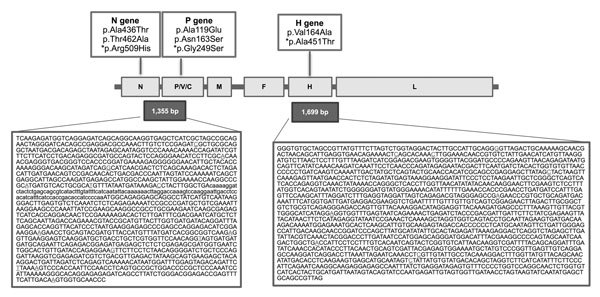
Genomic organization of the dolphin morbillivirus (GenBank accession no. KR337460) isolated from a newborn fin whale found stranded on Elba Island, Italy, October 2013. Boxes in first row indicate amino acid changes identified in each gene; asterisks indicate nonsynonymous amino acid substitutions. Boxes in the second row indicate the *morbillivirus* gene structure; horizontal lines indicate noncoding sequences. Boxes in the third row indicate the total length of the 2 analyzed virus fragments, and their sequences are shown, respectively, in boxes in the bottom row. N, nucleoprotein gene; P/V/C, phosphoprotein gene with nonstructural proteins V and C; H, hemagglutinin gene; F, fusion gene; L, large protein gene. Primers DMV-N1 and DMV-P2 (*11*) were used to amplify a 1,355-bp nt sequence (left box in bottom row) representing partial portion of N gene and P/V/C gene (shown in uppercase letters); lowercase letters indicate noncoding sequences. Three overlapping primer pairs were used to amplify a 1,699-bp nt sequence (right box in bottom row) representing the entire H gene. Underlined bases indicate nucleotide variations from the complete DMV sequence (GenBank accession no. AJ608288). The distribution of amino acid changes identified in each gene are shown in the top row.

Sequences from 5 lung and 4 cerebral plasmidic colonies were analyzed. Programs in the DNASTAR Lasergene software package (http://www.dnastar.com/t-dnastar-lasergene.aspx) were used to edit, assemble, and translate sequences. This technique enabled identification of a 1,355-bp DNA fragment from the newborn whale that encompassed partial nucleoprotein and phosphoprotein genes (i.e., the N1-P2 consensus fragment; GenBank accession no. KR337460). This fragment, from lung and cerebral samples, showed 98.89% sequence homology with the complete DMV genome (GenBank accession no. AJ608288) and 99.85% sequence homology with a DMV strain identified in long-finned pilot whales (*Globicephala melas*) that were affected by the 2006–2007 epidemic in the Mediterranean (GenBank accession no. HQ829972). Amino acid changes in KR337460, compared with AJ608288, are shown in Figure 2. The same DNA fragment was recovered from the newborn whale’s spleen and thymus by using the PCR protocol mentioned above (Technical Appendix Figure 2).

Viral hemagglutinin (H) protein mediates DMV entry into host cells by specifically binding with the whale's signaling lymphocyte activation molecule (SLAM)/CD150; thus, we also investigated variations in the H gene of the newborn whale by using the previously described cloning procedures for cerebral cDNA with 3 new overlapping primers pairs (Table 2; Figure 2). This technique enabled identification of a 1,699-bp DNA fragment encompassing a partial H gene sequence; 116 bp at the beginning of the gene were missing. The H consensus fragment, obtained from the cDNA clone for each primer pair, showed 99.41% sequence homology with the complete DMV genome (GenBank accession no. AJ608288) and 99.94% sequence homology with the DMV strain identified in long-finned pilot whales (GenBank accession no. HQ829972). We also identified 2 aa changes: the previously reported p.Val164Ala (*11*) and a novel variation, p.Ala451Thr, located within the H protein region (residues 382–582) involved in SLAM binding (Figure 2) (*12*). This variation was previously reported in other related morbilliviruses (*13*) and does not control any change in the tertiary structure of H antigen, as determined by using the SWISS-MODEL (http://swissmodel.expasy.org/) modeling program.

**Table 2:** Primers designed on dolphin morbillivirus isolate used for the total hemagglutinin gene sequence analysis of the virus detected in the newborn fin whale*

Primer	Nucleotide position	Sense sequence, 5′ → 3′	Fragment length, bp	
DMV-10F	7206–7226	GGGTGTGCTAGCCGTTATGT	718	
DMV-10R	7904–7924	TTCGTCCTCATCAATCACCA	718	
DMV 11F	7799–7891	CCGAACCTGATGATCCATTT	612	
DMV-11R	8391–8411	CGTAAATGTCCATCCCTGCT	612	
DMV-12F	8290–8309	AACCGGATCCCAGCTTATG	800	
DMV-12R	9070–9090	CCAGGTGCACTTCAGGGTAT	800	

*Isolate GenBank accession no. AJ608288. Annealing temperature for all primers was 56°C. DMV, dolphin morbillivirus; F, forward; R, reverse.

## Conclusions

The results of our direct (IHC and biomolecular) and indirect (serologic) testing provide evidence of DMV infection or exposure in 5 (55%) of 9 fin whales that were found stranded off the Mediterranean Coast during 2011–2013. These 5 infected whales correspond to 21.7% of the 23 whales stranded along the Italian coastline during 2006–2014. The other 4 examined whales showed no evidence of morbillivirus infection. The range of DMV-susceptible host species has progressively expanded (*5*), as highlighted by the recent report of DMV infection in a captive common seal (*Phoca vitulina*) during the 2011 outbreak (*14*). This expansion, combined with spread of DMV through the transplacental route, resulting in virus colonization of the thymus in fetuses, could represent DMV survival strategies among cetacean populations. In addition, our data argue in favor of an epidemic cluster of fatal DMV among the Mediterranean fin whale population, even though, on the basis of the amino acid sequence of the SLAM/CD150 viral receptor, this species is not included among those susceptible to DMV epidemics (*5*,*12*).

Although the single amino acid substitution, p.Ala451Thr, did not cause substantial variations in the structure of H antigen, the effect of the variation on protein functions is unclear. Recent studies showed that similar amino acid changes could affect virulence and infectivity of different *Canine distemper virus* (family *Paramyxoviridae*, genus *Morbillivirus*) strains, but such changes are often neutralized by compensatory mutations that preserve the biologic activity of H protein (*15*). Furthermore, despite the high sequence homology observed between N, P, and H genes of the DMV strain identified in the newborn fin whale in our study and in the isolates recovered from DMV-affected cetaceans during the 1990–1992 and the 2006–2007 epidemics (GenBank accession no. AJ608288), we cannot exclude that more prominent differences occurred in virus genes encoding for both structural and nonstructural proteins responsible for virulence and pathogenicity (e.g., P/V/C and fusion genes) (*5*); the simultaneous occurrence of primary structure differences, if any, in the SLAM/CD150 receptor should also be taken into account (*5*,*12*). In conclusion, although further studies are needed to elucidate the complex virus–host interaction dynamics and the putative influence exerted by environmental factors, DMV should be regarded as one of the major threats for the conservation of fin whales within the Mediterranean Sea.

**Technical Appendix.** Fin whale stranding sites in the Mediterranean Sea, 2006–2013, and agarose gel electrophoresis results of PCR for *Morbillivirus* with dolphin morbillivirus (DMV)–N1 and DMV-P2 primers.
