# Supplementary material for: Mediterranean Fin Whales (Balaenoptera physalus) Threatened by Dolphin MorbilliVirus
Source: Emerg Infect Dis. 2016 Feb;22(2):302–5. doi: 10.3201/eid2202.150882 (PMC4734534; doi:10.3201/eid2202.150882)
Supplement: Supplementary file 1 — Technical Appendix. Fin whale stranding sites in the Mediterranean Sea, 2006–2013, and agarose gel electrophoresis results of PCR for Morbillivirus with dolphin morbillivirus (DMV)–N1 and DMV-P2 primers. [file 15-0882-Techapp-s1.pdf]

Article DOI: http://dx.doi.org/10.3201/eid2202.150882

Mediterranean Fin Whales (*Balaenoptera physalus*) Threatened by Dolphin Morbillivirus

Technical Appendix

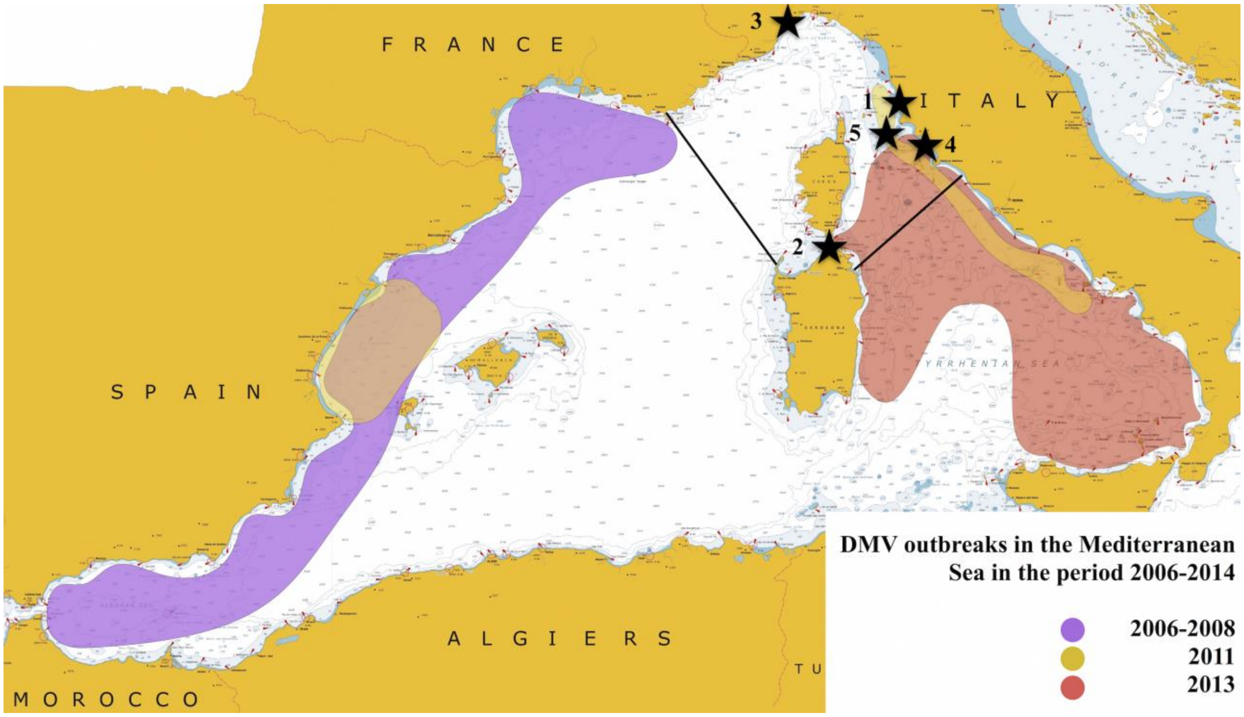

Technical Appendix Figure 1. Fin whale stranding sites (indicated by stars), compared with the distribution of dolphin morbillivirus (DMV) outbreaks that occurred in the western Mediterranean during 2006–2014.

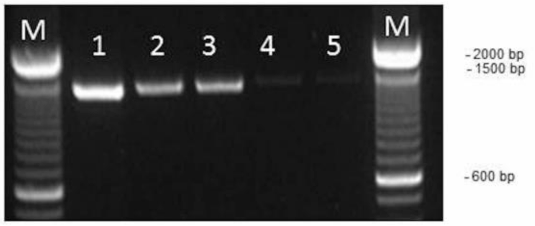

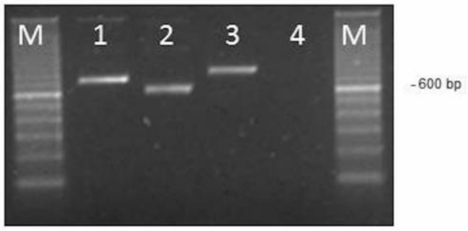

Technical Appendix Figure 2. The left agarose gel is the result of PCR for *Morbillivirus* with DMV-N1 and DMV-P2 primers (*1*). The PCR results do not represent a quantitative analysis for the amount of virus in different tissues. Lane M: DNA marker 100-bp DNA Ladder (Invitrogen); Lane 1: positive control (dolphin morbillivirus); Lane 2: Positive lung tissue sample from *Balaenoptera physalus*; Lane 3: positive brain tissue sample from *B. physalus*; Lane 4: positive spleen tissue sample from *B. physalus*; Lane 5: positive thymus tissue sample from *B. physalus*. The right agarose gel is the result of PCR amplification for *Morbillivirus* H gene using 3 new primers overlapping pairs described in Table 2. Lane M: DNA marker 100-bp (DNA Ladder; Invitrogen); lane 1: positive brain tissue sample from *B. physalus* (DMV-10F and DMV-10R); lane 2: positive brain tissue sample from *B. physalus* (DMV-11F and DMV-11R); lane 3: positive brain tissue sample from *B. physalus* (DMV-12F and DMV-12R); and lane 4: PCR-negative control.

Reference

1. Bellière EN, Esperón F, Sánchez-Vizcaíno JM. Genetic comparison among dolphin morbillivirus in the 1990–1992 and 2006–2008 Mediterranean outbreaks. Infect Genet Evol. 2011;11:1913–20. PubMed
